# A multilevel analysis of the determinants of missed opportunities for vaccination among children attending primary healthcare facilities in Kano, Nigeria: Findings from the pre-implementation phase of a collaborative quality improvement programme

**DOI:** 10.1371/journal.pone.0218572

**Published:** 2019-07-10

**Authors:** Abdu A. Adamu, Olalekan A. Uthman, Muktar A. Gadanya, Olatunji O. Adetokunboh, Charles S. Wiysonge

**Affiliations:** 1 Cochrane South Africa, South African Medical Research Council, Tygerberg, South Africa; 2 Centre for Evidence-based Health Care, Division of Epidemiology and Biostatistics, Department of Global Health, Faculty of Medicine and Health Sciences, Stellenbosch University, Cape Town, South Africa; 3 Warwick-Centre for Applied Health Research and Delivery (WCAHRD), Division of Health Sciences, University of Warwick Medical School, Coventry, United Kingdom; 4 Department of Community Medicine, Bayero University/Aminu Kano Teaching Hospital, Kano State, Nigeria; 5 School of Public Health and Family Medicine, University of Cape Town, Cape Town, South Africa; University of Ghana College of Health Sciences, GHANA

## Abstract

**Background:**

We aimed to determine the factors that are responsible for missed opportunities for vaccination (MOV) among children aged 0–23 months attending primary health care (PHC) facilities in Nassarawa, Kano State, Nigeria.

**Methods:**

This cross-sectional study was conducted in the pre-implementation phase of a quality improvement programme. One-stage cluster sampling technique was employed. Data were collected from caregivers of children aged 0–23 months in ten randomly selected PHC facilities in Nassarawa Local Government Area of Kano State. Semi-structured, interviewer administered questionnaires were used. Frequencies and percentages were used to summarize the data. Multilevel logistic regression model with fixed effect and random effect component was fitted to obtain measures of association and variation respectively.

**Results:**

Caregivers of 675 children responded. Among these children, the prevalence of MOV (for at least one antigen) was 36.15%. MOV (for individual antigens) was highest for inactivated polio vaccine followed by measles vaccine. The random effect model yielded an intraclass correlation coefficient of 9.60% for the empty model. The fixed effect model revealed that MOV was more likely among children that were accompanying a caregiver to the health facility (OR = 2.86, 95%CrI: 1.28 to 5.80) compared to those that were visiting the health facility for medical consultation. Failure to receive vaccination on the day of health facility visit (OR = 2.32, 95%CrI: 1.12 to 4.12) and visiting a clinic with three or more vaccinators (OR = 12.91, 95%CrI: 4.82 to 27.14) increased the likelihood of MOV.

**Conclusion:**

The study identified important local factors that are responsible for MOV which can be addressed in the QI programme.

## Introduction

Vaccines can improve the health of children and increase life expectancy by reducing the burden of death and disability caused by common infectious diseases [1, 2]. In order to extend this benefit to all children, Nigeria established an Expanded Programme on Immunization (EPI) [3]. Currently, the programme provides routine immunization with the following vaccines: Bacillus Calmette-Guerin (BCG), hepatitis B vaccine, oral polio vaccine (OPV), pentavalent vaccine (Penta), pneumococcal conjugate vaccine (PCV), inactivated polio vaccine (IPV), measles vaccine and yellow fever vaccine [4]. These vaccines are provided within the first year of life according to the national immunization schedule as follows: at birth (BCG, OPV0, HEPB0), at six weeks of age (Penta1, OPV 1, PCV1), at 10 weeks of age (Penta2, OPV2, PCV2), at 14 weeks of age (Penta3, OPV3, PCV3, IPV), and at nine months of age (measles and yellow fever) [4]. However, a significant number of children in the country are still unimmunized and full childhood immunization coverage is suboptimal [3, 5–7]. Even within the country, there is disparity in coverage level between geopolitical zones, with the North West zone reporting full immunization coverage level of 8% compared to 50% in the South West [8]. In Kano, which is one of the states in the North West zone, full immunization coverage is only 10% and coverage with the third dose of pentavalent vaccine is 16% [8]. Kano is highly populated, and the persistently poor coverage level has made it a high-risk state for polio transmission [9, 10]. Several factors contribute to low immunization coverage among which are missed opportunities for vaccination (MOV) in health service settings [11–14].

MOV refers to any contact with health services by an unvaccinated or partially vaccinated child (who is free of contraindications) which does not result in the child receiving all the recommended vaccine doses for their age according to the national schedule [11, 15]. Studies conducted in tertiary hospitals in Benin, Anambra and Enugu reported MOV prevalence of 27.6%, 17% and 15.1% respectively [16–18]. Furthermore, the level of “missed opportunities” for specific antigens also vary across settings. In Enugu and Benin, measles vaccines were the most commonly missed [16, 17].

**Fig 1:**
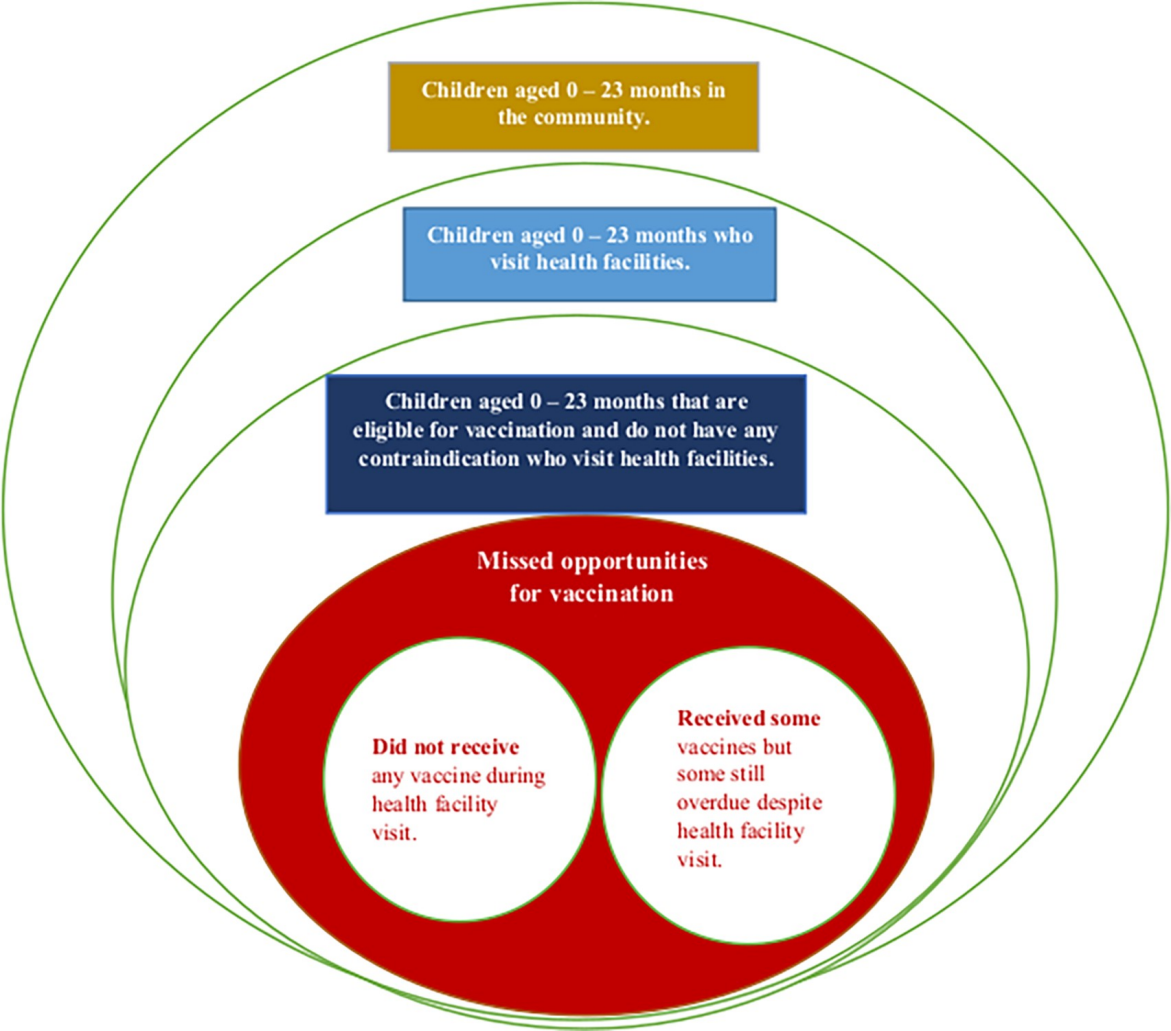
Euler diagram of missed opportunities for vaccination.

To standardize the procedure for quantifying MOV, the World Health Organization (WHO) built on an existing protocol to develop an updated MOV methodology [19, 20]. In the current MOV strategy, assessments are focused on children aged 0–23 months [19, 20]. The procedure involves interviewing caregivers while exiting health facilities and obtaining the immunization history of children from their home-based records (HBR) [21]. As illustrated in **Fig 1**, a “missed child” either didn’t receive any vaccine or received only some of their recommended vaccine doses.

Understanding the magnitude and factors that are responsible for MOV among children aged 0–23 months is relevant for practice and policy and can inform the development of interventions. A recent systematic review and meta-analysis, which included three studies from Nigeria (conducted in the South East and South South geopolitical zones) estimated the pooled prevalence of MOV among African children aged 0–23 months to be 27.26% [22]. In addition, the review highlighted several determinants of MOV and importantly, depicted the complexity of the problem by showing that factors are interrelated and interdependent using a causal loop diagram [22]. However, only 20 studies from 14 African countries were included [22]. So far, there is limited evidence from Kano, despite being a low immunization coverage setting.

In this study, the prevalence of MOV and its determinants were explored among caregivers attending primary health care (PHC) facilities in Kano State, Nigeria. This was to generate context-specific information that can be used by local immunization stakeholders and health workers in a quality improvement (QI) programme. Quality improvement (QI) is an approach for instituting rapid change in health systems through continuous effort and experiential learning in order to improve health outcomes [23]. It can be used to redesign health delivery systems like immunization services to improve uptake and reduce MOV [24].

Existing literature suggests that MOV occur in facilities where individual level factors originating from children and caregivers co-occur with health systems factors that affect immunization service delivery [22]. Since the influence of these contextual predictors differ across setting, the magnitude of MOV can vary, with resultant clustering effect in facilities. To explore this, a multilevel modeling framework was adopted. Conceptually, individuals (child and caregiver) were considered to be nested in health facilities. In line with this assumption, the determinants of MOV were also categorized into two, namely; individual- and health facility-related factors. The selection of these factors was informed by previous studies as well as background knowledge of the context [21, 22]. A conceptual framework is attached as **S1 Fig**.

This study focused on primary healthcare facilities because this level of healthcare is closest to people and communities [25]. It also has immunization as part of its key service components [25].

The specific objective was to determine the factors responsible for missed opportunities for vaccination among children aged 0–23 months attending primary healthcare facilities in Nassarawa, Kano State.

## Materials and methods

### Study design

A cross-sectional study design was used [26]. This was conducted in the pre-implementation phase of a collaborative QI programme. This observational study design enabled the measurement of the burden of MOV and its determinants at a specific point in time thus providing a snapshot of the phenomenon [26].

### Study setting

The study was conducted in Nassarawa LGA, which is one of the metropolitan LGAs in Kano [27]. This LGA has an area of 35km^2^ with a high prevalence of slum settlements [28]. According to the 2006 National Housing and Population Census, the population of Nassarawa was 596,669, with an estimated annual growth rate of 3.3% [29, 30]. The 2018 projected population of the LGA was 880,922. In addition, the projected population of children under one year of age and under five years of age are 35,236 and 176,184 respectively. Nassarawa LGA is further subdivided into 11 administrative wards. There are 18 public primary health care (PHC) facilities in the LGA that offer immunization services. According to the current minimum standards for primary health care (PHC) in Nigeria, these primary health care facilities are classified into primary health centers, primary health clinics, and health posts [25].

### Study population

Children aged 0–23 months who were brought to public PHC facilities in Nassarawa LGA by a caregiver (aged 18 years and above) were included in this study. In situations where a caregiver came to the health facility with more than one child, only the youngest child was considered to avoid overrepresentation.

### Sampling

Study participants were drawn from ten randomly selected public PHC facilities that provide immunization services in the LGA. One-stage cluster sampling technique was used. Each public primary health care facility was considered as a cluster. Within each cluster, all children aged 0–23 months who were brought to the facility by an eligible and consenting caregiver were selected.

### Sample size

The required sample size of children aged 0–23 months was 675. This was computed using Cochran’s equation for sample size, and based on the following assumptions: critical value of 1.96 (at 95% confidence level), a prevalence of MOV of 32.2% from a previous study, an accepted margin of error of 5%, non-response rate of 20% and design effect of 1.5 [15, 31, 32]. Design effect (Deff) was considered in order to account for clustering as respondents are embedded within specific primary health care facilities [33].

### Data collection

Data was collected using an interviewer administered semi-structured questionnaire. This questionnaire was adapted from WHO’s caregiver quantitative data collection tool as specified in the methodology for the assessment of MOV [19, 20]. The caregiver tool has already been pilot tested by WHO [34]. Before commencing data collection, the questionnaire was translated into Hausa Language and both versions were pre-tested in Kano Municipal and Kumbotso to ensure clarity and suitability of questions. Advocacy visits were paid to state and local government immunization stakeholders. This was to seek their buy-in and solicit for collaboration throughout the QI process. A one-day training of data collectors was conducted. During the training, each item on the questionnaire was discussed to ensure common understanding. Repeated dry runs were performed in both languages to improve their familiarity with the tools. Ethical considerations were also discussed. Face-to-face health facility exit interviews that were conducted between December 17–21, 2018 was used for this study. Data collection was usually between 8:00AM and 4:00PM and on weekdays only. The caregivers of all eligible children attending the PHC facilities were interviewed. Interviews were conducted by the trained data collectors in either English or Hausa Language depending on the preference of the respondent. The trained data collectors were fluent in both languages. The number of participants included per site is attached as **S1 Table**. After collecting information from the caregiver, the child’s immunization history was then obtained from their home-based record (HBR) also called “vaccination card” in the setting or any temporary vaccination document. The data collectors did not have any prior training on immunization. Research electronic data capture (REDCap) was used for collecting and managing the data collected for this study [35].

### Variables

Outcome variables: MOV for at least one antigen was used as the main outcome variable. This was a binary variable coded as *1*,*0* for MOV and no MOV respectively.

Explanatory variable: The explanatory variables were grouped into two levels as follows:

Level 1: child’s age group, child’s sex, birth order, reason for child’s visit to health facility, caregiver’s age, caregiver’s sex, marital status, relationship with child, occupation, level of education, duration from home to health facility, exposure to media messages about immunization, ever vaccinated child, ever refused immunization in health facility, vaccination card checked during this visit, knowledge of vaccines child needs and child vaccinated today.

Level 2: type of primary health care facility (primary health care clinic and primary health care center) number of health workers, number of vaccinators, location characteristics and electricity supply.

### Data analysis

The frequency and percentage of children with MOV (for at least one antigen) were calculated. Also, frequencies and percentages of MOV for each antigen were calculated. To account for the effect of clustering, *surveyset* command in Stata was specified before calculations [36, 37]. All the explanatory variables (individual and health services-related factors) were summarized using frequencies and percentages. Since clustered data were collected, assumption of independence would not hold. Therefore, to obtain correct standard errors for the measures of association between individual and health facility-related factors and MOV, as well as between-PHC facility variance, a multilevel logistic regression model was used [38]. Multilevel models are an extension of generalized linear models which address non-independence in data by generating cluster-specific random models [39]. In this model, we regarded individuals (children and caregivers) as level 1 and considers them as nested in primary health care facilities (level 2) [40].

In total, four models were built. In model 1, only health facility random intercept was included to estimate between-facility variance, thus the probability of MOV in this model was only a function of the health facility that a child attended. Model 2 included only individual-related factors (level one explanatory variables), and model 3, included only health facility-related factors. Finally, model 4, which is the full model, controlled for both individual and health facility-related factors. The models were fitted using Markov Chain Monte Carlo (MCMC) method [41]. In this method, a Markov chain makes successive selections of subsets of parameters from their posterior distributions [41]. The estimation setting was inputted manually to achieve a burn-in period of 10,000 iterations followed by a monitoring period of 5000 iterations [41]. Odds ratios with corresponding 95% credible intervals (CrI) were reported for the fixed effects. While for the random effect, variance, intraclass correlation coefficients (ICC) and mean odds ratios (MOR) were reported to quantify the influence of context. ICC was presented as percentages to represent the total variance in the probability of MOV that is accounted for by health facilities. While MOR represented total variance in the probability of MOV that is attributed to health facilities in the odds ratio scale. The deviance information criterion (DIC) was used to assess the model fit [42]. Lower DIC indicated a better fit [42]. Models were built in MLwiN version 3.01 from Stata 14.2 using *runmlwin* command [41].

### Ethical approval

Ethical clearance for this study (with reference number: S18/02/044) was obtained from Stellenbosch University Health Research Ethics Committee. Also, the study was approved by research ethics committees at Kano State Ministry of Health (with reference number: MOH/Off/797/T.I/374) and Aminu Kano Teaching Hospital (with reference number: NHREC/21/08/2008/AKTH/EC/2296). An information sheet was read to respondents and written informed consent was obtained. The study participants were informed that they could choose not to answer any question or leave the study at any time. No identifiers were collected to ensure anonymity.

## Results

The total number of children aged 0–23 months included in this study were 675. Caregivers of children were interviewed across all ten primary healthcare facilities in Nassarawa LGA, Kano.

### Burden of MOV

The prevalence of MOV (for at least one antigen) among children aged 0–23 months attending primary healthcare facilities in Nassarawa LGA was 36.15%. The prevalence of MOV for inactivated polio vaccine (IPV) and measles vaccines were 45.10% and 43.28% respectively. The prevalence of MOV for all the other antigens are shown in **Table 1**.

**Table 1:** Prevalence of missed opportunities for vaccination (MOV) among children aged 0–23 months attending primary healthcare facilities in Nassarawa LGA, Kano.

Variables	Total (N)	Frequency (n)	Percentage (%)
**MOV for one or more antigens**			
MOV (1+)	675	244	36.15
**MOV for each dose of antigen**			
Bacillus Calmette-Guerin (BCG)	670	23	3.43
Hepatitis B Vaccine (HBV)	667	58	8.70
Birth Dose Oral Polio Vaccine (OPV0)	667	48	7.20
First Dose Oral Polio Vaccine (OPV1)	475	91	19.16
Second Dose Oral Polio Vaccine (OPV2)	365	103	28.22
Third Dose Oral Polio Vaccine (OPV3)	286	115	40.21
First Dose Pentavalent Vaccine (PENTA1)	470	102	21.70
Second Dose Pentavalent Vaccine (PENTA2)	368	106	28.80
Third Dose Pentavalent Vaccine (PENTA3)	281	110	39.15
First Dose Pneumococcal Conjugate Vaccine (PCV1)	475	106	22.32
Second Dose Pneumococcal Conjugate Vaccine (PCV2)	369	114	30.89
Third Dose Pneumococcal Conjugate Vaccine (PCV3)	287	120	41.81
Inactivated Polio Vaccine (IPV)	286	129	45.10
Measles Vaccine (MCV)	134	58	43.28
Yellow Fever Vaccine (YFV)	135	56	41.48

MOV1+ = missed opportunities for vaccination for at least one antigen

A total of 589 children in this study were aged 0–11 months, while 86 were aged 12–23 months. Among all the children, 52.83% were males. The commonest reason for bringing children to the health facility was for vaccination. Most caregivers were females and 55.85% of caregivers completed secondary education. Majority of children have ever been vaccinated before and 86.76% of caregivers said they know the vaccines that their children require. Other characteristics are shown in **Table 2**.

**Table 2:** Characteristics of children aged 0–23 months and their caregivers attending primary health care facilities in Nassarawa LGA, Kano.

	Total Frequency		MOV*	
			Yes	No
Variables	Number (n)	Percentage (%)	Number (n)	Number (n)
**INDIVIDUAL-LEVEL FACTORS**		** **		
**Child’s age group**				
0–11 months	589	87.26	200	389
12–23 months	86	12.74	44	42
**Child's sex**				
Male	355	52.83	121	234
Female	317	47.17	122	195
**Birth order**				
First child	176	26.07	52	124
Second child	135	20.00	58	77
Third child and above	364	53.93	134	230
**Reason for child’s visit to health facility**				
Medical consultation or hospitalization	170	25.26	84	86
Vaccination	386	57.36	106	280
Only accompanying caregiver	64	9.51	39	25
Newborn or growth and development clinic	53	7.88	15	38
**Caregiver's age group**				
18–24 years	261	38.67	101	160
25–31 years	276	40.89	97	179
>31 years	138	20.44	46	92
**Caregiver's sex**				
Male	29	4.31	17	12
Female	644	95.69	225	419
**Marital status**				
Married	648	96	234	414
Unmarried	27	4	10	17
**Occupation**				
Housewife	547	82.01	194	353
Employed	99	14.84	37	62
Student	21	3.15	8	13
**Level of education**				
No formal education or didn’t complete primary school	101	14.96	33	68
Completed primary school	113	16.74	46	67
Completed secondary school	377	55.85	133	244
Post-secondary education	84	12.44	32	52
**Duration from caregiver home to health facility**				
Within 30 minutes	648	96.00	241	407
More than 30 minutes	27	4.00	3	24
**Exposure to media messages about immunization in the last month**				
Yes	583	86.76	209	374
No	89	13.24	34	55
**Ever vaccinated child**				
Yes	655	97.76	233	422
No	15	2.24	10	5
**Ever refused immunization in health facility**				
Yes	14	2.07	10	4
No	661	97.93	234	427
**Vaccination card checked during this visit**				
Yes	537	80.03	183	354
No	134	19.97	60	74
**Knowledge of vaccines child needs**				
Yes	583	86.76	204	379
No	47	6.99	20	27
Not sure	42	6.25	19	23
**Child vaccinated today**				
Yes	443	66.22	127	316
No	226	33.78	115	111

*MOV = Missed opportunities for vaccination

The percentage of children who attended a primary health center was 74.22%, while 25.78% attended a primary health clinic. Majority of the health facilities have more than 12 health workers. Also, majority have at least three vaccinators. Other characteristics of the health facilities are shown in **Table 3**.

**Table 3:** Characteristics of public primary health care facilities that provide immunization services in Nassarawa LGA, Kano.

	Total Frequency		MOV*	
			Yes	No
Variables	Number (n)	Percentage (%)	Number (n)	Number (n)
**HEALTH FACILITY-LEVEL FACTORS**		** **		
**Type of primary health facility**				
Primary health centre	501	74.22	168	333
Primary health clinic	174	25.78	76	98
				
**Number of health workers**				
Less than 12	54	8.00	23	31
12 or more	621	92.00	216	405
				
**Number of vaccinators**				
Less than 3	126	18.67	26	100
3 or more	549	81.33	218	331
**Location characteristics**				
Slum	365	54.07	128	237
Non-slum	310	45.93	116	194
**Electricity supply**				
None	153	22.67	67	86
1hours - 8hours	305	45.19	116	189
More than 8 hours	217	32.15	61	156

*MOV = Missed opportunities for vaccination

### Factors associated with missed opportunities for vaccination

Measure of association: The OR with Crl for covariates in each model are shown in Table 4. Model 4 which adjusted for all covariates revealed that reason for health facility visit, duration from home to health facility, vaccination on day of visit, and number of vaccinators in health facilities were associated with MOV. Children who were only accompanying a caregiver to the health facility were more likely to have MOV compared to those who were visiting for medical consultation or hospitalization (OR = 2.86, 95%CrI: 1.28 to 5.80). MOV was less likely in those who were visiting the health facility for vaccination (OR = 0.47, 95%CrI: 0.23 to 0.85) or attending newborn growth and development clinic (OR = 0.40, 95%CrI: 0.16 to 0.79) compared to those who were visiting the hospital for medical consultation. MOV was also less likely among children of caregiver who reported that the duration from their home to the health facility was more than 30 minutes (OR = 0.16, 95%CrI: 0.02 to 0.48). Children who didn’t received vaccination on the day of contact with the health facility were more likely to have MOV compared to those who received vaccination (OR = 2.32, 95%CrI: 1.12 to 4.12). Children attending facilities with at least three vaccinators had more likelihood of MOV (OR = 12.91, 95%CrI: 4.82 to 27.14). Odd Ratios for other variables for each model are shown in **Table 4**.

**Table 4:** Factors associated with missed opportunities for vaccination among children aged 0–23 months attending primary healthcare facilities in Nassarawa LGA, Kano.

	Model 1		Model 2		Model 3		Model 4	
	OR (95%CrI)	p-value	OR (95%CrI)	p-value	OR (95%CrI)	p-value	OR (95%CrI)	p-value
**FIXED-EFFECT**								
**INDIVIDUAL-LEVEL FACTORS**								
**Child’s age group**								
0–11 months			ref				ref	
12–23 months	-		1.76 (0.96–3.02)	0.04	-		1.65 (0.90–2.77)	0.06
**Child's sex**								
Male			ref				ref	
Female	-		1.27 (0.87–1.80)	0.12	-		1.28 (0.85–1.85)	0.13
**Birth order**								
First child			ref				ref	
Second child	-		1.92 (1.03–3.25)	0.02	-		1.82 (1.00–3.01)	0.03
Third child and above	-		1.81 (0.97–2.96)	0.03	-		1.74 (0.93–2.98)	0.04
**Reason for child’s visit to health facility**								
Medical consultation or hospitalization			ref				ref	
Vaccination	-		0.50 (0.27–0.85)	0.01	-		0.47 (0.23–0.85)	0.01
Only accompanying caregiver	-		2.70 (1.18–5.36)	0.01	-		2.86 (1.28–5.80)	0.00
Newborn or growth and development clinic	-		0.42 (0.17–0.84)	0.01			0.40 (0.16–0.79)	0.01
**Caregiver's age group**								
18–24 years			ref				ref	
25–31 years	-		0.67 (0.40–1.04)	0.04	-		0.67 (0.40–1.07)	0.05
>31 years	-		0.82 (0.43–1.45)	0.22	-		0.85 (0.42–1.56)	0.25
**Caregiver's sex**								
Male			ref				ref	
Female	-		0.49 (0.15–1.09)	0.04	-		0.50 (0.19–1.22)	0.05
**Marital status**								
Married			ref				ref	
Unmarried	-		0.88 (0.30–1.20)	0.30	-		0.85 (0.29–1.95)	0.29
**Occupation**								
Housewife			ref				ref	
Employed	-		0.94 (0.27–2.80)	0.37	-		0.88 (0.40–1.67)	0.30
Student	-		1.05 (0.63–2.45)	0.42	-		1.01 (0.26–2.65)	0.40
**Level of education**								
No formal education or didn’t complete primary school			ref				ref	
Completed primary school	-		1.31 (0.63–2.45)	0.28	-		1.22 (0.57–2.27)	0.35
Completed secondary school	-		1.14 (0.60–2.03)	0.40	-		1.07 (0.53–1.88)	0.47
Post-secondary education	-		1.09 (0.40–2.48)	0.49	-		1.02 (0.36–2.37)	0.42
**Duration from caregiver home to health facility**								
Within 30 minutes			ref				ref	
More than 30 minutes	-		0.17 (0.03–0.47)	0.001	-		0.16 (0.02–0.48)	0.00
**Exposed to media messages about immunization in the last month**								
Yes			ref				ref	
No	-		1.42 (0.73–2.50)	0.16	-		1.20 (0.64–2.10)	0.34
**Ever vaccinated child**								
Yes			ref				ref	
No	-		2.95 (0.66–9.20)	0.10	-		2.81 (0.68–8.85)	0.11
**Ever refused immunization in health facility**								
Yes			ref				ref	
No	-		0.47 (0.08–1.46)	0.08	-		0.43 (0.09–1.27)	0.06
**Vaccination card checked during this visit**								
Yes			ref				ref	
No	-		0.86 (0.46–1.50)	0.25	-		0.89 (0.48–1.53)	0.29
**Knowledge of vaccines child needs**								
Yes			ref				ref	
No	-		1.33 (0.61–2.51)	0.27	-		1.36 (0.62–2.55)	0.27
Not sure	-		1.75 (0.74–3.55)	0.13	-		1.79 (0.69–3.71)	0.11
**Child vaccinated today**								
Yes			ref				ref	
No	-		2.18 (1.12–3.90)	0.01	-		2.32 (1.12–4.12)	0.02
**HEALTH FACILITY-LEVEL FACTORS**								
**Type of primary health facility**								
Primary health centre					ref		ref	
Primary health clinic	-				2.58 (1.12–7.04)	0.01	1.98 (0.94–4.17)	0.04
**Number of health workers**								
Less than 12					ref		ref	
12 or more	-				1.96 (0.59–5.19)	0.14	2.90 (0.76–6.92)	0.071
**Number of vaccinators**								
Less than 3					ref		ref	
3 or more	-				4.56 (2.12–10.55)	0.00	12.91 (4.82–27.14)	0.00
**Location characteristics**								
Slum					ref		ref	
Non-slum	-				1.20 (0.31–2.41)	0.38	1.44 (0.54–3.63)	0.32
**Electricity supply**								
None					ref		ref	
1hours - 8hours	-				2.61 (0.74–13.87)	0.11	1.99 (0.68–4.66)	0.12
More than 8 hours	-				0.66 (0.31–1.40)	0.07	0.76 (0.32–1.62)	0.17

Model 1 –Empty model with only random intercept

Model 2 –Individual level covariates only

Model 3 –Health facility level covariates only

Model 4 –Full model with all individual and health facility level covariates

OR = Odds ratio; CrI = Credible Interval

Measure of variation: On Table 5, model one (empty model) showed that there is variation in the log-odds of MOV across the 10 primary healthcare facilities (0.35, 95%CrI: 0.09 to 1.02), with an intraclass correlation (ICC) of 9.60%. This ICC indicates that the variance in odds of MOV could be attributed to health facilities, thus suggesting the influence of context. The MOR in model 1–4 are 1.76, 2.07, 1.37 and 1.31 respectively. This further confirms the presence of contextual phenomenon in these primary health care facilities. The DIC for Model 4 is 796.18.

**Table 5:** Random effect estimates of missed opportunities for vaccination across public primary healthcare facilities in Nassarawa LGA, Kano.

	Model 1	Model 2	Model 3	Model 4
**RANDOM-EFFECT**				
**Health facility-level**				
Variance (95%CrI)	0.35 (0.09–1.02)	0.58 (0.16–1.66)	0.11 (0.00–0.81)	0.08 (0.00–0.61)
ICC (%)	9.60	15.00	3.20	2.40
MOR (%)	1.76	2.07	1.37	1.31
Explained variation (%)				
**Model fit statistics**				
DIC	851.41	797.55	851.03	796.18

Model 1 –Empty model with only random intercept

Model 2 –Individual level covariates only

Model 3 –Health facility level covariates only

Model 4 –Full model with all individual and health facility level covariates

OR = Odds ratio; CrI = Credible Interval; ICC = Intraclass correlation; MOR = Mean odds ratio; DIC = Deviance Information Criteria;

## Discussion

This current study included 675 children aged 0–23 months from 10 PHC facilities in Nassarawa LGA, Kano. MOV prevalence was 36.15% among children attending these PHC facilities. MOV for specific antigens was highest for IPV at 45.10%, followed by measles vaccine at 43.28%. Factors such as visiting facility for vaccination, accompanying a caregiver to facility, attending newborn, growth and development care, duration from home to health facility more than 30 minutes, receiving vaccination on day of clinic visit, and having three or more vaccinators were found to be associated with MOV. “Facility context” influenced the occurrence of MOV as ICC was found to be 9.60% in the empty model.

### Limitations and strengths

Some limitations and strengths should be considered when interpreting the findings from this study. As a cross-sectional study, MOV and associated factors were assessed at the same time therefore assuming a cause–effect relationship may not be appropriate. Data was collected from caregivers using exit interviews in health facilities, as such, they may give socially acceptable responses thus leading to social desirability bias. Although data were clustered, multilevel analysis technique was used to model the effect of these clusters. In addition, the model that accounted for the effect of clusters was treated as a random effect model thus improving the generalizability to other PHC facilities in the local government area. Also, immunization history was obtained from home-based records thus improving the accuracy of our MOV estimates.

### Missed opportunities for vaccination in Nassarawa, Kano

Immunization is an essential evidence-based intervention that should be provided to all children who needs it upon contact with health facilities [43]. Although immunization uptake was found to be high among children who visited the PHC facilities where this study was conducted, many eligible children still do not receive all the recommended vaccines or vaccines doses for their age. In this study, we found an MOV prevalence of 36.15%. This is higher than previously reported prevalence level in other studies that were conducted in Nigeria [16–18]. This might be due to difference in the level of healthcare. The current study focused on primary health care level, while earlier studies sampled children in tertiary health facilities [16–18]. Another important consideration is the overall immunization coverage in the area. The states were previous studies were conducted had higher full immunization coverage level compared to where this present study was conducted [8]. Regarding specific antigens, MOV was highest for IPV, followed by measles, then PCV3, yellow fever vaccine, OPV3 and PENTA3. In some previous studies, measles was reported to be the highest [16, 17]. A possible explanation for why MOV was highest for these vaccines might be because they are among the last vaccines in the series and are given to older children [14]. In line with WHO’s recommended methodology, in this study, we only included those that are in possession of their home-based records [19]. To obtain quality and reliable information about a child’s immunization history, the home-based records is required [44]. This study advanced existing knowledge by employing multilevel modeling approach to study MOV. The multilevel analysis technique demonstrated that facility context influence MOV occurrence. This evidence highlights the need for local immunization stakeholder and health workers to prioritize strategies that promotes the use of context-specific, tailored interventions to address MOV.

### Implications for the quality improvement programme in Nassarawa, Kano

Based on the MOV planning guide, assessments only constitute the initial steps in the broader MOV strategy [20]. The information that are generated from facilities are to be used for improving them through follow-up interventions to reduce MOV and improve immunization coverage [20]. This is why the MOV strategy is also considered an immunization strategy [20]. Similarly, in this study, the MOV assessment was conducted as part of a quality improvement programme to generate information that can be used to inform the selection of locally relevant change ideas for improving the PHC facilities. This bottom-up approach is recommended by the World Health Organization [20].

The probability of MOV occurring among children who are only accompanying a caregiver to the health facility was found to be high. Although visiting a health facility for the purpose of accompanying a caregiver invariably constitute contact with health services, health workers might be reluctant to pay attention to accompanying children thus resulting in MOV. Furthermore, children who weren’t provided vaccination on the day of visit were more likely to experience MOV. These two factors underscores the need for the QI programme to broaden its scope beyond just the immunization system to the entire PHC service delivery system. Service delivery should be re-designed such that immunization services can be offered daily and screening of HBR is strengthened across all service delivery points. This can improve access to immunization for all child users of health services in the PHC facilities as well as accompanying children. Since majority of the caregivers are females, and PHC facilities offer services like family planning and antenatal care, these points should be prioritized in the QI programme. This can go together with a re-orientation exercise for health workers to sensitize them on the need to reduce MOV. MOV was less likely among those who reported that the duration from their home to the PHC facility was more than 30 minutes. Paradoxically, children who visit facilities with higher number of vaccinators were more likely to experience MOV.

### Implications for Broader Policy

Descriptive analysis showed that MOV occurred in more than half of children aged 12–23 months. And although the estimate was imprecise, the confidence interval for the association between children in their second year of life and MOV after adjusting for other covariates included some considerable likelihood of occurrence (OR = 1.65, 95%CrI: 0.90–2.77). This should not be ignored. The second year of life can be an important period for catch-up immunization in this setting especially for children that had earlier missed their vaccination. Therefore, these is a need for health policy makers to begin to consider policies that will institutionalize immunization within this age group.

Given the low immunization coverage level in this setting, the state primary health care management board (PHCMB) might need to consider integrating MOV assessments into the health system as a routine process to monitor this important child health quality problem and empower health workers in PHC facilities to act accordingly. This can serve as a form of “surveillance and response” mechanism that tracks and immunize unvaccinated and partially vaccinated children who make contact with facilities. Also, policy makers at the primary health care management board and ministry of health should include plans to reduce MOV into broader health sector plans to enable its consideration in the various vertical interventions that are implemented in primary health care facilities.

### Implications for future research

This cross-sectional study highlighted that MOV is an important problem in this setting, however, the assessment was conducted in only 10 primary health care facilities in one urban LGA. Therefore, there are still several unanswered questions about the dynamics of MOV in this setting that need to be explored. Using the Evidence Population Intervention Comparison Outcome and Time stamp (EPICOT+) framework, recommendations for future research were proposed as shown on Table 6 [45]. There is need for more MOV assessment in Kano, specifically, and North West Nigeria, in general. Assessments should be conducted in PHC facilities as well as other levels of health care to enable more robust understanding of this immunization sub-system problem. Furthermore, assessments should span urban and rural localities. In addition, future MOV assessments in specialized clinics like sickle cell diseases clinics, pediatric HIV clinics among others are warranted. As recommended in the planning guide, assessments should go hand in hand with site-specific interventions that can reduce MOV.

**Table 6:** Use of EPICOT+ framework to recommend future research based on gaps in evidence.

Element	Recommendation(s)
**Core elements**	
Evidence (State of evidence)	Paucity of MOV assessments in Kano particularly, and North West Nigeria
Population (Population of interest)	MOV assessments using WHO’s methodology among the following: a. Children aged 0–23 months attending general hospitals b. Children aged 0–23 months attending primary health care facilities in metropolitan local government areas. c. Children aged 0–23 months attending primary health care facilities in rural areas. d. Children aged 0–23 months attending specialized clinics
Interventions	Tailored interventions implemented through a. Facility-based quality improvement b. Collaborative quality improvement
Comparisons	Control health facilities
Outcomes	Proportion of MOV
Time stamp	January 2019
**Optional element**	
Study type	Cross sectional surveys

## Conclusion

This study demonstrated that quantitative methods are a useful tool for identifying potential areas for intervention in a collaborative QI programme for addressing MOV in PHC facilities. Focusing on the recommended age group as specified in the updated MOV methodology streamlined data collection and target group for intervention that is aligned with the interest of immunization stakeholders. A key lesson from this study was the critical role of stakeholder engagement, particularly because it was for a QI programme. As efforts to meet target coverage level intensifies, we hope that local immunization stakeholders will integrate the MOV strategy into routine health systems processes.

## Supporting information

S1 Fig Multilevel conceptual framework of the determinants of missed opportunities for vaccination among children aged 0–23 months attending primary healthcare facilities in Nassarawa, Kano State. (DOCX)

S1 Table Number of participants selected per facility during data collection in primary health care facilities in Nassarawa LGA, Kano. (DOCX)
