# Supplementary material for: A multilevel analysis of the determinants of missed opportunities for vaccination among children attending primary healthcare facilities in Kano, Nigeria: Findings from the pre-implementation phase of a collaborative quality improvement programme
Source: PLoS One. 2019 Jul 10;14(7):e0218572. doi: 10.1371/journal.pone.0218572 (PMC6619653; doi:10.1371/journal.pone.0218572)
Supplement: S1 Fig — (DOCX) [file pone.0218572.s001.docx]

**HEALTH FACILITY-RELATED FACTORS**

**Facility characteristics:** type of health facility, number of health workers, number of vaccinators, location characteristics and electricity supply

Remain unvaccinated or under-vaccinated **despite contact with health service**

Missed opportunities for vaccination (MOV)

**Child characteristics**: Age, sex, birth order and reason for health facility visit

**Caregiver demographic characteristics:** Age, sex, marital status, relationship with child, level of education, occupation

**Caregiver access to immunization services:** Duration from home to health facility, ever vaccinated child, knowledge of vaccines child needs, child vaccinated today, vaccination card checked during visit, and ever refused immunization services.

**Caregiver media access:** Exposure to media messages about immunization

**INDIVIDUAL-RELATED FACTORS**
