# Supplementary material for: A multilevel analysis of the determinants of missed opportunities for vaccination among children attending primary healthcare facilities in Kano, Nigeria: Findings from the pre-implementation phase of a collaborative quality improvement programme
Source: PLoS One. 2019 Jul 10;14(7):e0218572. doi: 10.1371/journal.pone.0218572 (PMC6619653; doi:10.1371/journal.pone.0218572)
Supplement: S1 Table — (DOCX) [file pone.0218572.s002.docx]

S1 Table: Number of participants selected per facility during data collection in primary health care facilities in Nassarawa LGA, Kano

| Facility | Number of Participants |
| --- | --- |
| PHC 1 | 74 |
| PHC 2 | 65 |
| PHC 3 | 56 |
| PHC 4 | 54 |
| PHC 5 | 86 |
| PHC 6 | 55 |
| PHC 7 | 44 |
| PHC 8 | 49 |
| PHC 9 | 66 |
| PHC 10 | 126 |
